# Multi-targeting chemoprevention of Chinese herb formula Yanghe Huayan decoction on experimentally induced mammary tumorigenesis

**DOI:** 10.1186/s12906-019-2456-1

**Published:** 2019-02-13

**Authors:** Jingwei Li, Xiaofei Liu, Hongzhi Chen, Ziyuan Sun, Hanhan Chen, Lei Wang, Xiaohui Sun, Xiangqi Li

**Affiliations:** 1 Department of breast surgery, Affiliated Hospital of Shandong University of Traditional Chinese Medicine, Jinan, 250014 Shandong China; 2 Shandong University of Traditional Chinese Medicine, Jinan, 250335 Shandong China; 3 Affiliated Hospital of Taishan Medical University, Taian, 271000 Shandong China

**Keywords:** Chemoprevention, Mammary tumorigenesis, Multi-components, Multi-targeting, Chinese herb formula

## Abstract

**Background:**

Development of safe and effective chemopreventive agents is a winning strategy in reducing the morbidity and mortality of breast cancer. The current study was to investigate the mechanism-based chemopreventive potential of a Chinese herb formula Yanghe Huayan (YHHY) Decoction on the classical 7,12-dimethylbenz(a)anthracene (DMBA) induced rat mammary carcinogenesis model.

**Methods:**

Female Sprague-Dawley rats at 42 days of age were orally administered with a human equivalent dose of YHHY Decoction at 0.02 ml/g (10 mg/ml) once daily, starting 1 wk. before and 4 wks following DMBA treatment. Mammary tumor occurrence was monitored every day. The length of time before palpable tumor is examined is defined as tumor-free survival time. High performance liquid chromatography (HPLC) analyses were adopted to identify major chemical compositions of the decoction. Following bioinformatics data mining and experimental analyses were performed to demonstrate the underlying mechanism of action.

**Results:**

DMBA animals receiving YHHY Decoction exhibited a significant delay (*P* = 0.014) and in some animals prevention (*P* = 0.046) of tumor occurrence without obvious toxicity. Oncogenic myc activation was significantly suppressed in the DMBA-induced rats by the YHHY treatment. Eight major chemical compositions of the decoction were identified and were shown to interfere with multiple tumorigenic pathways simultaneously in the mammary tumors, including inducing tumor apoptosis and up-regulating pro-apoptotic protein Bax and down-regulating anti-apoptotic protein Bcl-2; suppressing abnormal cell proliferation and the MAPK/ERK, PI3K/AKT signalings; blocking neo-angiogenesis and the VEGF/KDR signaling, and inhibiting oxidative stress in the mammary tumors.

**Conclusion:**

The multi-components and multi-targeting properties of the YHHY Decoction support its use as a potent chemopreventive drug in breast cancer.

## Background

The prevalence of mammography screening, and the routine use of large core needle biopsies for the diagnosis of clinically occult breast lesions have led to an increased detection of early precursor lesions or precancerous breast conditions, such as atypical lobular hyperplasia (ALH), atypical ductal hyperplasia (ADH), lobular carcinoma in situ (LCIS) and ductal carcinoma in situ (DCIS) [1]. Several large cohort studies have agreed that the risk of breast cancer in women with ALH, ADH and LCIS is about 4–8 times higher than that of the general population [2–5]. ALH, ADH and LCIS are thought to be reversible, and the efficacy of chemoprevention with tamoxifen, raloxifene, and exemestane has been established in large prospective trials [6–9]. However, concerns about serious side effects preclude the widespread and long-term use of these medical options [10, 11]. On average, only 4% of high-risk women decide to take these chemopreventive drugs [12]. DCIS is referred as “stage zero breast cancer” and it has become one of the most commonly diagnosed breast conditions in recent years. Appropriate treatment for DCIS is crucial to prevent invasive breast cancer. However, although nearly all conceivable combinations of surgery, radiotherapy and systemic treatments have been tested in different trials, the situation is still unsatisfactory [13]. Hence, new breast cancer chemopreventive agents with acceptable efficacy and little toxicity that suitable for long-term use are in urgent need.

Traditional Chinese Medicine (TCM), with thousands of years clinical practice history, has been widely used for disease prevention and management, and is gaining popularity worldwide for promoting healthcare [14–16]. The advantages of “multi-component, multi-target and multi-pathway” combinational regulatory mechanism and relative low toxicity make the TCM herb formula promising for new drug development [17, 18]. We have used the Yanghe Huayan (YHHY) Decoction, which was originally derived from “*Wai Ke Yi Jing”* (Qing Dynasty) more than a century ago, to treat patients with chronic breast fibrosis and palpable lump in our clinic, and the average curative rate, defined as complete resolution or marked improvement of lumps and pain for at least two months with the herbal treatment, was 90% (560 women)(unpublished data). The YHHY Decoction is an aqueous preparation of herbal mixture and consists mainly of extracts from 8 Chinese medicinal herbs: Lu Jiao Jiao (*Cornu Cervi Pantotrichum, CCP),* Tu Beimu *(Rhizoma bolbostemmae, RB)*, Bai Jie Zi (*Semen brassicae, SB*), Rou Gui (*Cinnamomum cassia, CC*), Pao Jiang (*Baked ginger, BG*), Ma Huang (*Ephdra vulgaris, EV*), Hu Tao Rou (*Juglans regia L, JRL*), and Sheng Gan Cao (*Glycyrrhiza uralensis, GU*). Although several individual herbs in the formula have been studied to have anti-tumor activity on cancer cell lines or tumor models [19–23], clinical benefits of these components on breast cancer prevention have ever been achieved only when mixing them together.

The current study investigated the underlying mechanism of the formula cocktail on preventing tumorigenesis in chemical carcinogen DMBA induced mammary tumors. Environmental chemical carcinogen exposure, majorly the polycyclic aromatic hydrocarbon chemicals (PAH), plays a significant role in causing breast cancer as evidenced by many epidemiological and laboratory studies [24, 25]. DMBA is a prototypical PAH that has been used extensively to induce mammary carcinomas in female Sprague-Dawley (SD) rats to study the process of carcinogenesis. Features of this process that make the model comparable to human disease include similarities in histologic progression, hormone dependence, angiogenic phenotype and oncogenic signaling activation [26–28]. In our study, YHHY Decoction was given to the animals starting 1 wk. before and 4 wks following DMBA induction as a chemopreventive regimen. Our results from both “dry” lab bioinformatics data mining and “wet” lab experimental analyses demonstrated that the YHHY Decoction exerted chemopreventive efficacy in the DMBA model of breast cancer by targeting multiple tumorigenic pathways simultaneously.

## Methods

### Preparation of YHHY decoction

The YHHY Decoction is an aqueous preparation from 8 Chinese medicinal herbs: *CCP* 15 g*, RB* 9 g, *SB* 6 g, *CC* 3 g, *BG* 3 g, *EV* 9 g, *JRL* 6 g, and *GU* 6 g. The raw herb materials were purchased from Bozhou Huqiao Pharmaceutical Co Ltd. (Anhui, China) in September 2015, and were authenticated by Dr. Feng Li at the Department of Pharmacognosy, Shandong University of TCM. A voucher specimen was deposited at TCM Pharmacy, Affiliated Hospital of Shandong University of TCM, with the voucher numbers as *CCP* 1508160116, *RB* 1507180227, *SB* 1507270896, *CC* 1508030119, *BG* 1506080417, *EV* 1507210325, *JRL* 1508200021, and *GU* 1507290632. Each raw herb was homogenized to fine powder and the eight herbs were mixed thoroughly. A 8.4 g sample of the mixed fine-ground powder was accurately weighted and extracted with 84 mL of boiling H_2_O for 6 h and then centrifuged at 12,000 rpm for 15 min, both the volatile oil and the supernatant were collected. The pellet was resolved in 60% ethanol (1:1.5 volume) and statically stewed for 24 h. The mixture was then centrifuged at 12,000 rpm for 15 min, and the supernatant was collected. The two supernatants were combined and dried in the rotary evaporator at 160 rpm at 30 °C. Then the dried powder was accurately weighed and dissolved in the volatile oil Tween-80 solution (1:1 volume) at 1 mg/ml.

### Chemicals and reagents

DMBA was purchased from Sigma-Aldrich (St. Louis, MO). Primary antibodies, c-myc (NCL-cMYC, RRID:AB_563665, from Leica Microsystems), phosphor-c-myc (PA5–35814, RRID:AB_2553124, from Thermo Fisher Scientific), CD-105/Endoglin (NCL-CD105, RRID:AB_563482, from Leica Microsystems), and Ki-67 (sc-23,900, RRID:AB_627859), Bax (sc-70,407, RRID:AB_1119412), Bcl-2 (sc-7382, RRID:AB_626736), ERK1/2 (sc-135,900, RRID:AB_2141283), pERK1/2 (sc-136,521, RRID:AB_10856869), PI3K (sc-365,290, RRID:AB_10846944), AKT (sc-5298, RRID:AB_626658), KDR (sc-101,559, RRID:AB_1123212) and β-actin (sc-47,778, RRID:AB_2714189) from Santa Cruz Biotechnology (Santa Cruz, CA) were used. Antioxidant assay kit (cs0790) and In Situ Cell Death Detection Kit (11,684,817,910 ROCHE) were purchased from Sigma-Aldrich (St. Louis, MO). Ventana Basic DAB (3,3-diaminobenzidine) Detection kit was from Boster Biotechnology, Wuhan, China.

### Animals

The animal study was conducted upon the approval by the Institutional Animal Care and Use Committee of Shandong University of TCM (SDUTCM2012042001). All experiments were performed in accordance with relevant guidelines and regulations. Pathogen-free virgin female Sprague-Dawley rats (MGI Cat# 5651135, RRID:MGI:5651135), approximately 42 days of age, weight 150 ± 10 g, were provided by Experimental Animal Center of Shandong University of TCM and housed in an animal facility accredited by the Chinese Association for the Accreditation of Laboratory Animal Care. The rats were acclimatized to standard housing conditions, including ambient temperature of 22 ± 2 °C, relative humidity at 30–50%, and a 12-h light-dark cycle, in plastic cages (maximum 4 animals/cage) for 1 wk. before initiation of the experiment. The animals had free access to the nutrition formula rodent diet (NTP-2000 standard) and drinking water.

### Animal treatment protocol

The potential chemopreventive role of YHHY Decoction was investigated using a well-established DMBA-induced rat mammary tumorigenesis model [27, 29]. Following 1-wk acclimatization period, the rats were randomly divided into 4 groups with *n* = 6 per each group: group A-normal control with vehicle treatment; group B-DMBA induction with vehicle treatment, group C-DMBA induction with YHHY Decoction treatment and group D-normal control with YHHY Decoction treatment. The experiments were independently repeated three times, so the final sample size for each group is *n* = 18 rats. YHHY Decoction was fed through oral gavage (p.o.) once daily for 5 wks (1 wk. before and 4 wk. following DMBA treatment). A human equivalent dose of YHHY Decoction at 1 mg/ml, given by 0.02 ml/g body weight, was used based on the human to animal dose conversion formula (https://www.fda.gov/downloads/drugs/guidances/ucm078932.pdf, page 7). Following 1 wk. feeding with the YHHY Decoction, rats in the Groups B and C were administered a single dose of DMBA at 50 mg/kg body weight (dissolved in corn oil) by oral gavage to induce mammary carcinogenesis. This dose of DMBA was chosen so that substantial tumor incidence could be produced but not so high as to overwhelm chemopreventive action of YHHY Decoction. The specific time for DMBA exposure is based on previous carcinogenic bioassays indicating that rats at this age possess high frequency of terminal end buds that are sensitive to the established mammary carcinogen DMBA [30]. Rats in Group C were continually fed with YHHY Decoction for another 4 weeks after the DMBA administration (totally for 5 wks). Food, water intake and behavioral patterns were monitored daily, body weights were recorded twice a week. Palpation of mammary glands started 4 wks following DMBA treatment with a frequency of twice per week. The length of time before palpable tumor is examined is defined as tumor-free survival time. The experiments were terminated at 16 wks post-DMBA administration.

### Tissue harvest and histopathology analyses

Animals were euthanized by cervical dislocation after overnight fast. The mammary tumors were carefully excised from mammary gland parenchyma and rinsed with phosphate-buffered saline (PBS) (pH = 7.4). Each tumor was measured in 2 perpendicular directions by a caliper to obtain an average diameter, then was cut into 2 halves. One half was immediately snap frozen in liquid nitrogen and used for molecular analysis. The other half was fixed in 4% paraformaldehyde and used for histopathological and immunohistochemical analysis. Serial tumor sections at 10 μm thickness were prepared for hematoxylin and eosin (H&E) and relevant immunohistochemistry staining. The mammary tumors were classified according the established criteria [31].

### Immunohistochemical staining

Tumor sections were stained with primary Ki-67 antibody, and CD-105 antibody with the Ventana Basic DAB Detection kit for cell proliferation and microvessel detection. Apoptotic cells were detected by TUNEL reaction with In Situ Cell Death Detection Kit. The slides were evaluated by an independent pathologist who was blinded to group assignment and outcome assessment using a light microscope (BX41, Olympus). Five random fields under 20× objective for each slide, and at least 5 slides for each tumor sample were analyzed.

### Tissue lysate antioxidant capacity analysis

Similar as previous described [32], two sets of measurements were performed on the excised mammary tissues: (i) antioxidant enzyme activities – superoxide dismutase (SOD), glutathione peroxidase (GPx) and catalase (CAT); and (ii) biochemicals – malondialdehyde (MDA) and total nitrate (NOx). Each frozen mammary tissue was divided into two portions (each approximately 0.5 g). One portion was homogenized in 1.5% ice-cold KCl solution to give a 10% suspension and used for the MDA assay. The other portion was cut into several small pieces, homogenized in PBS (pH 7.4), with a *w*/*v* ratio of 1:5, and spun at 13,000×g for 15 min, at 4 °C. The supernatant was separated for the measurements of SOD, CAT, GPx activities and NOx levels. The protein level in the supernatant was determined spectrophotometrically by the method of Lowry et al. [33] using BSA as a standard. SOD activity was expressed as the amount causing 50% inhibition of the reduction of cytochrome c per milligram of protein (U/mg of protein), with bovine copper–zinc SOD (Cu/Zn SOD) as standard [34]. CAT activity was measured by the method of Luck [35]. The decomposition of the substrate H_2_O_2_ was monitored spectrophotometrically at 240 nm. Specific activity was defined as micromole substrate decomposed per minute per milligram of protein (expressed as U/mg protein). GPx activity was measured as before [36]. Specific GPx activity (U/mg protein) was calculated as micromole NADPH consumed per minute per milligram of protein using an appropriate molar absorption coefficient (6220/M per cm). The level of MDA, determined using the method of Mihara and Uchiyama [37], was expressed as nmol/mg protein. The level of NOx was measured by the method developed by Sastry et al. [38]. A calibration standard involving potassium nitrate was used to calculate the total concentrations of nitrate, which was expressed as μmol/mg protein.

### Western blot analysis

Equal quantities of protein from the tumor tissue lysate were processed for Western blotting. Each sample was denatured, electrophoresed, and transferred onto a PVDF membrane. After blocking the membrane, blots were incubated in specific primary antibodies and then secondary antibodies following the manufacturer’s instructions. Densitometric analysis was conducted using ImageJ software. The primary antibodies used include: anti-c-myc (1:5000), anti-phospho-c-myc (0.5 μg/ml), anti-Bax (1:1000), anti-Bcl-2 (1:2000), anti-ERK1/2 (1:500), anti-pERK1/2 (1:500), anti-PI3K (1:1000), anti-AKT (1:800), anti-KDR (1:400) and anti-β-actin (1:2000). No grouping of gels/blots cropped from different parts of the same gel or from different gels, fields, or exposures was performed.

### HPLC analyses

The reference standards of eight chemical constituents, including adenosine, uracil, tubeimoside A, allyl isothiocyanate, trans-cinnamic acid, cinnamic aldehyde, 6-shogaol and polysaccharides (purity≥98%) were purchased from Chengdu Biopurify Phytochemicals Ltd., (Chengdu, China). Methanol, acetonitrile (Fisher Scientific, USA), and acetic acid (Tianjin Chemical Regent Co., Ltd., Tianjin, China) were of HPLC grade. The distilled water was obtained from Wahaha Co., Ltd. (Hangzhou, China).

An Agilent 1100 liquid chromatography system was used for the analysis, equipped with a diode array detector working in the range of 190-400 nm, a quaternary solvent delivery system, a column temperature controller, and an autosampler. The chromatographic data was recorded and processed with Agilent Chromatographic Work Station software.

For HPLC analysis and quantification of each constituents, 1 mg/ml decoction was filtered through a membrane filter (0.45 μm pore size) prior to injection and analyzed three times. Adenosine and uracil, cinnamic acid and cinnamaldehyde were examined simultaneously in one aliquot of the decoction sample, and other constituents were examined individually. Previously validated chromatographic conditions were applied to detect the concentrations of each constituent [39–45]:

For simultaneously detection of adenosine and uracil analysis, C18 analytical column (TIANHE Kromasil C18, 4.6 mm × 250 mm, 5 μm) was used. The mobile phase consisted of water (A)-acetonitrile (B); A:B was as follows: o min, 99:1; 5 min, 98:2; 10 min, 98:2; 20 min, 96:4; 42 min, 45:55; 55 min, 40:60; 60 min, 0:100. The flow rate and column temperature were set constantly at 0.3 ml/min and 30 °C. The detection wavelength was at 260 nm.

For simultaneously detection of cinnamic acid and cinnamaldehyde, C18 analytical column (Gemini C18, 4.6 mm × 250 mm, 5 μm) was used. The mobile phase consisted of solvent A (1.0%, *v*/v, aqueous acetic acid) and solvent B (1.0%, v/v, acetic acid in acetonitrile). The gradient flow of A:B was as follows: 0 min, 95:5; 40 min, 30:70; 45 min, 0:100, hold for 5 min; 55 min, 95:5, hold for 15 min. The flow rate and column temperature were set constantly at 1 ml/min and 40 °C. The detection wavelength was at 280 nm.

For detection of tuberimoside A, C18 analytical column (RP C18, 4.6 mm × 250 mm, 5 μm) was used. The mobile phase consisted of methanol-water (V:V = 68:32). The flow rate and column temperature were set constantly at 0.1 ml/min and 25 °C. The detection wavelength was at 214 nm.

For detection of allyl isothiocyanate, sphereclone ODS-2 column (4.6 mm × 15cmm, 5 μm) was used. The mobile phase was acetonitrile. The flow rate and column temperature were set constantly at 0.1 ml/min and 25 °C. The detection wavelength was at 242 nm.

For detection of 6-shogaol, C18 analytical column (Alltima HP C18, 4.6 mm × 250 mm, 5 μm) was used. The mobile phase consisted of water (A) and acetonitrile (B). The A:B flow was as follows; 0 min, 55:45; 8 min, 50:50; 17 min, 35:65; 32 min, 0:100 B, 38 min, 0:100, 43 min, 55:45, 48 min, 55:45. The flow rate and column temperature were set constantly at 1 ml/min and 30 °C. The detection wavelength was at 230 nm.

The reference standards were accurately weighed and dissolved in 60% ethanol, and then diluted to appropriate concentration ranges for the establishment of calibration curves. All stock and working standard solutions were stored in brown bottles at 4 °C until used for analysis.

### Data mining using ingenuity pathway analysis (IPA)

Except tubeimosides A, other seven chemical constituents of the YHHY Decoction were found in the IPA database (Qiagen, USA), (RRID: SCR_008653) and their direct interacting molecules and endogenous chemicals, totally 293, were added to the analysis list. Core Analysis/Expression Analysis was performed using the Ingenuity Knowledge Base (RRID: SCR_008117) as a reference for *p* value calculation of the gene populations, and Experimentally Observed Direct Relationship was set to generate function networks.

### Statistical analysis

Cumulative percentage of animals with tumor occurrence was plotted against time (weeks). Data were analyzed using Kaplan–Maier Analysis followed by Log-Rank test. The incidence of mammary tumor development in various groups was analyzed by Fisher’s exact probability test. Other data are presented as mean ± SD. Significant differences among various groups were determined by two-sided student t test followed by Holm-Sidak test. *P* < 0.05 was considered to be statistically significant. The commercial software SigmaPlot 11.0 (Systat Software, Inc., San Jose, CA) was used for all statistical analysis.

## Results

### General observations

**Fig. 1:**
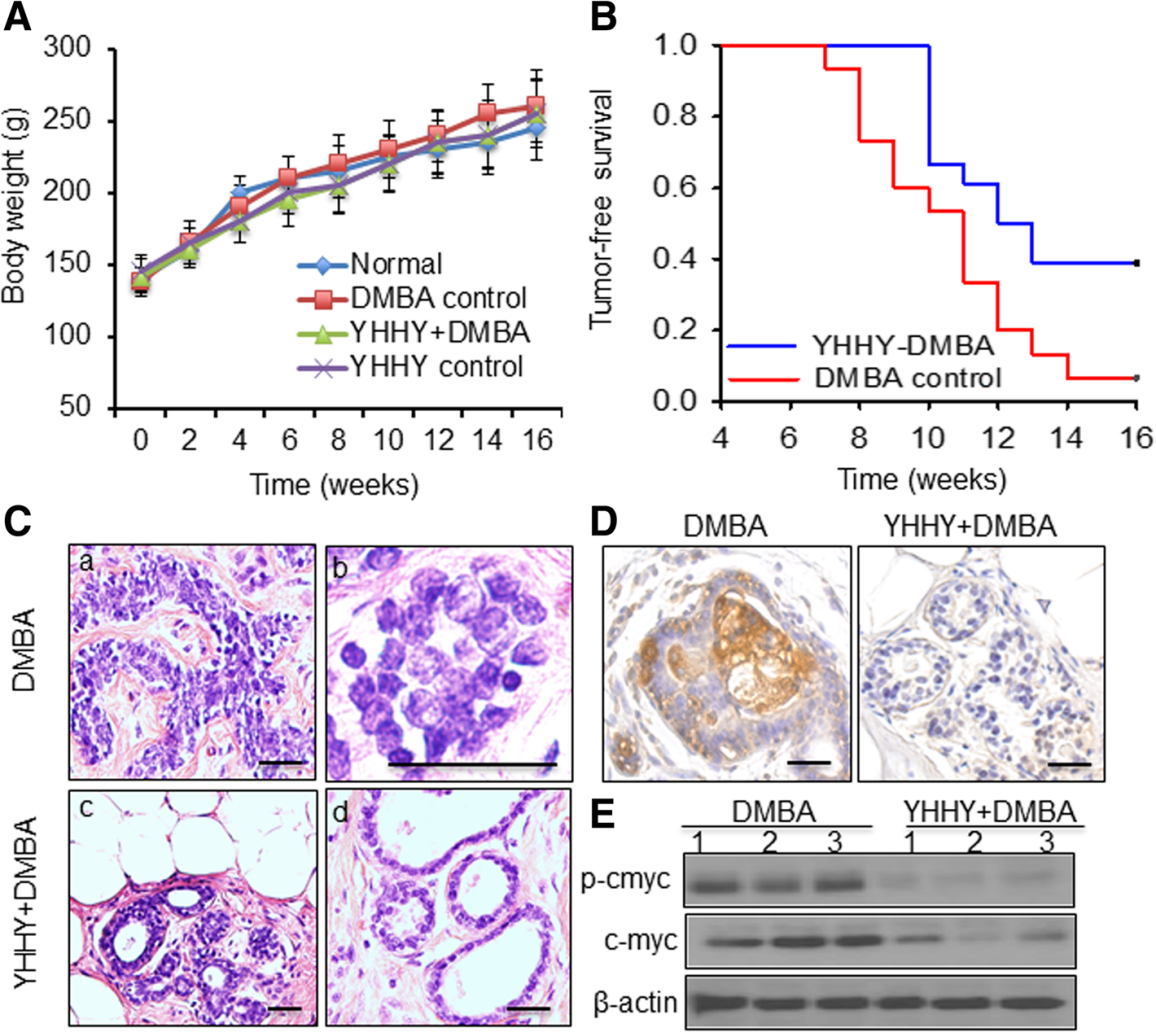
Effects of YHHY Decoction on DMBA-induced tumorigenesis and oncogenic myc activation. **a**. Effect of YHHY Decoction treatment on body weight gain during DMBA-induced mammary tumorigenesis in rats. Each data point indicates mean ± SD. There were 10–18 animals in various groups. No significant difference in body weights was observed among various rat groups at any time point. **b**. Effect of YHHY Decoction treatment on the occurrence of palpable tumors in the DMBA-induced rats. The length of time before palpable tumor is examined is defined as tumor-free survival time. *n* = 18 of the YHHY+DMBA group, and *n* = 14 of the DMBA control group. *P* = 0.014, Log rank test. **c**. Histopathology of the tumors detected in the DMBA control and YHHY Decoction treatment groups. DMBA control tumors showed extensive epithelial proliferation (**a**) and nuclear pleomorphism (**b**). Tumors treated with YHHY Decoction showed significantly improved cell architecture (**c**) and almost normal ductal and alveolar structure (**d**). Scale bar: 50 μm **d**. Immunohistochemical staining of phospho-c-myc in the mammary glands. Rats’ tissue were harvested two weeks after DMBA induction. The YHHY decoction treatment was initiated 1 week before DMBA induction and lasted for two weeks. Extensive staining of phospho-c-myc was observed in all examined animals in the DMBA group, while none of the animals in the YHHY prevention group showed obviously positive staining (*n* = 3). Scale bar: 50 μm **e**. Expressions of phospho-c-myc and total c-myc in 3 individual tumors in the YHHY Decoction treated vs. DMBA control group

The well-established DMBA-induced rat mammary tumorigenesis model was used in the current study [27, 29]. There were 4 animal groups: group A-normal control with vehicle treatment; group B-DMBA induction with vehicle treatment, group C-DMBA induction with YHHY Decoction treatment and group D-normal control with YHHY Decoction treatment. The rats received YHHY Decoction gained their weight at similar rates as the group A rats (Fig. 1a). Food and water intake was monitored periodically throughout the study and did not differ among the treatment groups. There were no observed behavioral changes among animal groups (data not shown).

### YHHY decoction inhibited DMBA-induced tumorigenesis and oncogenic myc activation

The development of palpable tumors was first observed in the DMBA control rats 7 wks after DMBA induction (Fig. 1b). In contrast, rats fed with YHHY Decoction did not develop mammary tumors until wk. 10 after DMBA treatment (*P* = 0.015, log-rank test). At the end of the study which was 16 wk. post DMBA induction, while 94% (17/18) rats in the vehicle group were detected with palpable tumors, there was only 61% (11/18) rats receiving YHHY Decoction were found to bear tumors (*P* = 0.041, fisher exact test). Power analysis showed that having 18 animals in each group provided 100% power to detect the increase of the mean tumor-free survival time from 10.788 ± 0.646 wks in the DMBA control group to 12.944 ± 0.646 wks in the YHHY+DMBA group with alpha = 5%. These results indicated that YHHY Decoction treatment not only delayed the tumor latency, but also prevented the occurrence of mammary tumors in some rats. Tumors in the YHHY treatment group also showed 74% less of the average weight than those in the DMBA controls (5.1 ± 1.7 g vs. 19.5 ± 4.5 g, *P* < 0.05).

Histopathological examination on the tumor sections indicated that tumors in the group B (DMBA control) rats showed extensive epithelial proliferation and enlargement of the alveolus as a fused glandular pattern (Fig. 1c), as well as a presence of nuclear pleomorphism (Fig. 1c). Epithelial cells demonstrated various nuclear sizes and irregular chromatin (Fig. 1c). The YHHY Decoction treatment improved the cellular architecture remarkably. Most of the tissue appeared normal ductal and alveolar structure without hyperplastic changes on epithelial cells (Fig. 1c).

The terminal end buds in mammary gland comprise epithelial stem cells that are sensitive to carcinogen DMBA for malignant transformation [46]. Since myc proto-oncogene is a hallmark of cancer initiation [47], we first examined the expression of phosphorylated c-myc protein in the mammary glands upon DMBA induction and YHHY prevention. The results showed that two weeks after DMBA induction the mammary epithelial cells already exhibited elevated expression of phospho-c-myc, but its expression was absent in all the examined animals in the YHHY group (*n* = 3) (Fig. 1d). Besides tumor initiation, sustained myc activation also contributes to autonomous tumor proliferation and growth [47]. When examining the c-myc expression in all the endpoint tumors in the two groups (Fig. 1e), the results showed a significant decrease in the levels of phosphor-c-myc in tumors of YHHY-treated rats vs. tumors of the control rats (526 ± 83 vs. 831 ± 126, in relative densitometric units, *P* < 0.05). The concentrations of total c-myc protein were also decreased dramatically in the tumors of YHHY-treated rats (834 ± 118 vs. 1215 ± 185 in relative densitometric units, *p* < 0.05).

### HPLC examination of eight chemical constituents in the YHHY decoction

HPLC analyses identified eight major chemical compositions of the decoction (6-shogaol, cinnamic acid, cinnamic aldehyde, polysaccharide, adenosine, uracil, tubeimoside A and allyl isothiocyanate) (Table. 1).

**Table. 1:** HPLC detection of eight major chemical compositions of the YHHY decoction.

Chemical constituents	Linear range (μg/ml)	Limit of Quantity (μg/ml)	Concentration (μg/ml)
6-shogaol	4.5–30	1.5	22.5 ± 3.5
cinnamic acid	2.3–300	0.06	3.5 ± 0.05
cinnamic aldehyde	1.6–105	0.03	61.5 ± 5.6
polysaccharide	1.5–75	0.05	30.2 ± 4.6
adenosine	2.8–115	0.02	18.9 ± 2.6
uracil	2.7–230	0.01	11.1 ± 2.1
tubeimoside A	0.4–200	0.01	27.2 ± 2.3
allyl isothiocyanate	0.2–105	0.1	20.1 ± 3.7

Data are expressed as mean ± SD (*n* = 3)

### Ingenuity pathway analysis (IPA) identified biological functions of the chemical constituents in YHHY decoction

The multifarious chemical constitutes of Chinese herbal cocktail may indicate complex mechanisms of action that warrant a systemic exploration. After searching in the IPA database, 7 out of the 8 chemicals were found and their direct-interacting molecules, totally 293 molecules, were used to analyze the disease or function networks related to the chemicals. Overall, the 293 molecules were significantly (*P* < 0.001) enriched in the top function networks of cell death and survival (apoptosis), cellular function and maintenance (cellular homeostasis), inflammatory response, organism injuries and abnormalities (inflammation of organ), free radical scavenging (synthesis of reactive oxygen species), cardiovascular system development and function (development of vasculature, angiogenesis), cell cycle (cell cycle progression), cellular movement (invasion of cells), and so on. Table. 2 listed the detailed category, disease and function annotation, *p* value and number of molecules for each network. Although needs further experimental validation on specific DMBA tumors, these results gave us an overall picture about the functions or mechanisms of the formula cocktail as a whole. In the following studies, we examined the activities of four major functions of the YHHY formula in the DMBA tumors.

**Table. 2:** Top function networks of the YHHY Decoction chemical components and their 293 interacting molecules in IPA

Categories	Disease or Function Annotation	*p*-Value	# Molecules
Cell Death and Survival	apoptosis	5.16E-25	59
Cellular Function and Maintenance	cellular homeostasis	1.91E-22	46
Tissue Morphology	Quantity of cells	1.1E-22	42
Cellular Development, Cellular Growth and Proliferation	Cell proliferation of tumor cell lines	3.38E-21	41
Inflammatory Response, Organism Injuries and Abnormalities	inflammation of organ	5.02E-27	38
Cardiovascular System Development and Function	development of vasculature, angiogenesis	9.15E-22	37
Free Radical Scavenging	synthesis of reactive oxygen species	4.2E-24	36
Cell Cycle	cell cycle progression	5.47E-14	31
Cellular Movement	invasion of cells	1.08E-18	31
Cancer, Cell Death and Survival, Tumor Morphology	Cell death of cancer cells	1.28E-24	29

### YHHY decoction induced tumor cell apoptosis, up-regulated Bax and down-regulated Bcl-2 expression

**Fig. 2:**
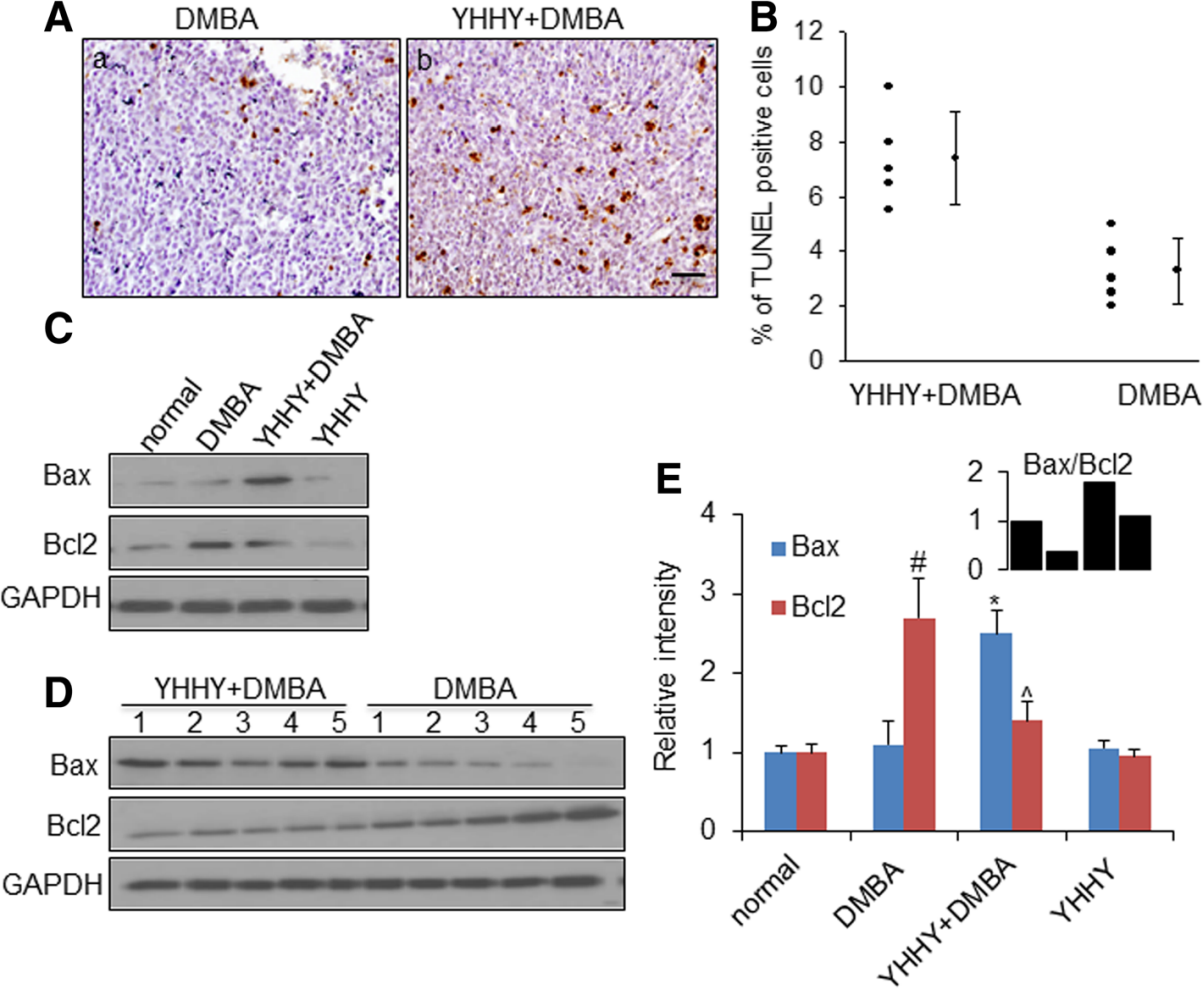
YHHY Decoction induced tumor cell apoptosis. **a**. Representative TUNEL staining images showing the apoptotic cells in tumor sections in **a**: DMBA-induced rat and b: YHHY treated DMBA rat. Apoptotic cells were detected by TUNEL reaction with In Situ Cell Death Detection Kit. TUNEL-positive cells contain dark-brown nuclei. Scale bar: 50 μm. **b**. Quantitative analysis of the % of TUNEL positive cells in the tumor sections. *P* = 0.0023, student t test. *n* = 5 animals per group. **c**. Representative Western blot images of Bax and Bcl2 in one tumor sample from each group. **d**. Expressions of Bax and Bcl2 in 5 individual tumors in the YHHY Decoction treated vs. DMBA control group. **e**. Quantification of the western blot analysis for Bax and Bcl2 in normal mammary glands (normal and YHHY groups) and mammary tumors (DMBA and YHHY+DMBA groups). # *p* < 0.05 vs. normal; * *p* < 0.05 vs. DMBA; ^ *p* < 0.05 vs. DMBA. The up-right corner graph showing the ratio of Bax/Bcl2 in each group

TUNEL assay was performed to evaluate the extent of cell apoptosis in the mammary tumor specimens. In the tumors from DMBA control rats, the presence of apoptotic cells was extremely rare. However, a significant increase of the apoptotic cell population was examined in the YHHY plus DMBA group (Fig. 2a, b).

To investigate the molecular signalings involved in the apoptosis-inducing effect of YHHY Decoction, Bax and Bcl2, two apoptosis-related proteins, both were in the 293 molecule list, were evaluated in the mammary tumors by Western blotting analysis. The Bax immuno-reactive band was detected extremely low in the DMBA control tumors (Fig. 2c, d). A significant increase of Bax expression was found in the tumors obtained from YHHY treatment group (Fig. 2c, d). The mammary tumors from DMBA control group showed extensive Bcl2 protein expression (Fig. 2c, d), and YHHY treatment exhibited considerable inhibition of Bcl2 expression (Fig. 2c, d). Oral administration of YHHY Decoction elevated the Bax/Bcl2 ratio under DMBA exposure (Fig. 2e). These data clearly indicated a pro-apoptotic mechanism of YHHY-mediated prevention of rat mammary tumorigenesis.

### YHHY decoction inhibited abnormal tumor cell proliferation and the MAPK/ERK, PI3K/AKT signalings

**Fig. 3:**
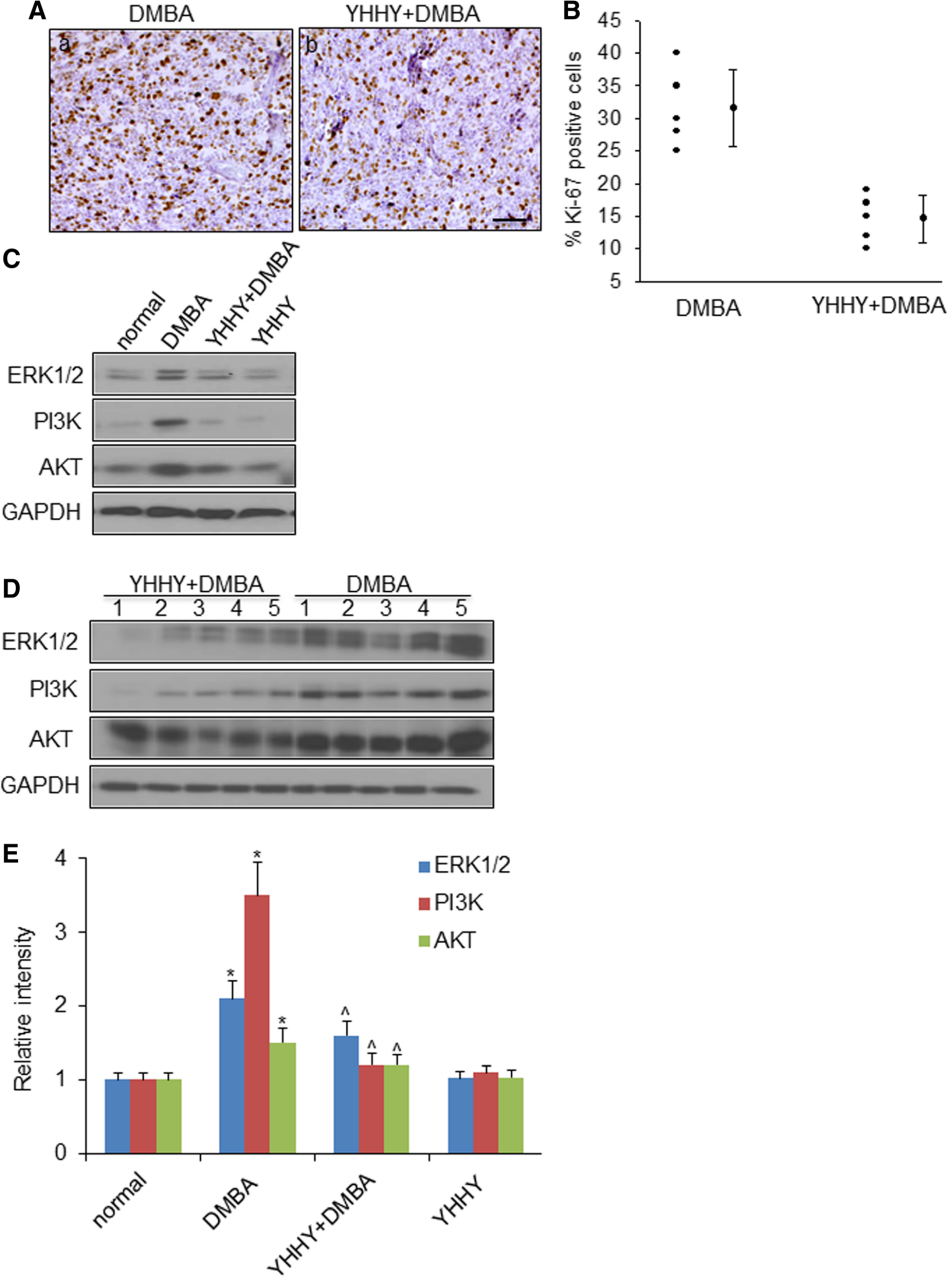
YHHY Decoction inhibited tumor cell proliferation. **a**. Representative images of Ki-67 immunohistochemistry staining showing the proliferative cells in tumor sections in a: DMBA-induced rat and b: YHHY treated DMBA rat. Tumor sections were stained with primary Ki-67 antibody and the DAB Detection kit for cell proliferation detection. Ki-67-positive cells contain dark-brown nuclei. Scale bar: 50 μm. **b**. Quantitative analysis of the % of Ki-67 positive cells in the tumor sections. *P* < 0.05, student t test. *n* = 5 animals per group. **c**. Representative Western blot images of ERK1/2, PI3K and AKT in one tumor sample from each group. **d**. Expressions of ERK1/2, PI3K and AKT in 5 individual tumors in the YHHY Decoction treated vs. DMBA control group. **e**. Quantification of the western blot analysis for ERK1/2, PI3K and AKT in normal mammary glands (normal and YHHY groups) and mammary tumors (DMBA and YHHY+DMBA groups). * *p* < 0.05 vs. normal; ^ *p* < 0.05 vs. DMBA

To determine whether YHHY Decoction impacted cell proliferation in the DMBA-induced mammary tumors, immunohistochemical staining of Ki-67 was assessed in the tumor sections. An abundant Ki-67-positive cells were found in the tumors from DMBA control rats, indicating active cell proliferation. YHHY Decoction suppressed cell proliferation to 30% of the DMBA tumors (Fig. 3a, b).

The activation of MAPK/ERK and PI3K/AKT signalings has been reported to contribute to both tumor initiation and oncogenic phenotype of mammary tumors through the effects on cell proliferation [1, 48]. ERK and AKT were both identified as the interacting molecules among the 293 molecules of the chemicals. Expressions of total ERK1/2, PI3K and AKT were examined to be 2–3 folds up-regulated in the DMBA control tumors compared to the normal mammary glands, and YHHY Decoction significantly suppressed their expressions (Fig. 3c, d).

### YHHY decoction blocked neoangiogenesis and inhibited the VEGF/KDR signaling

**Fig. 4:**
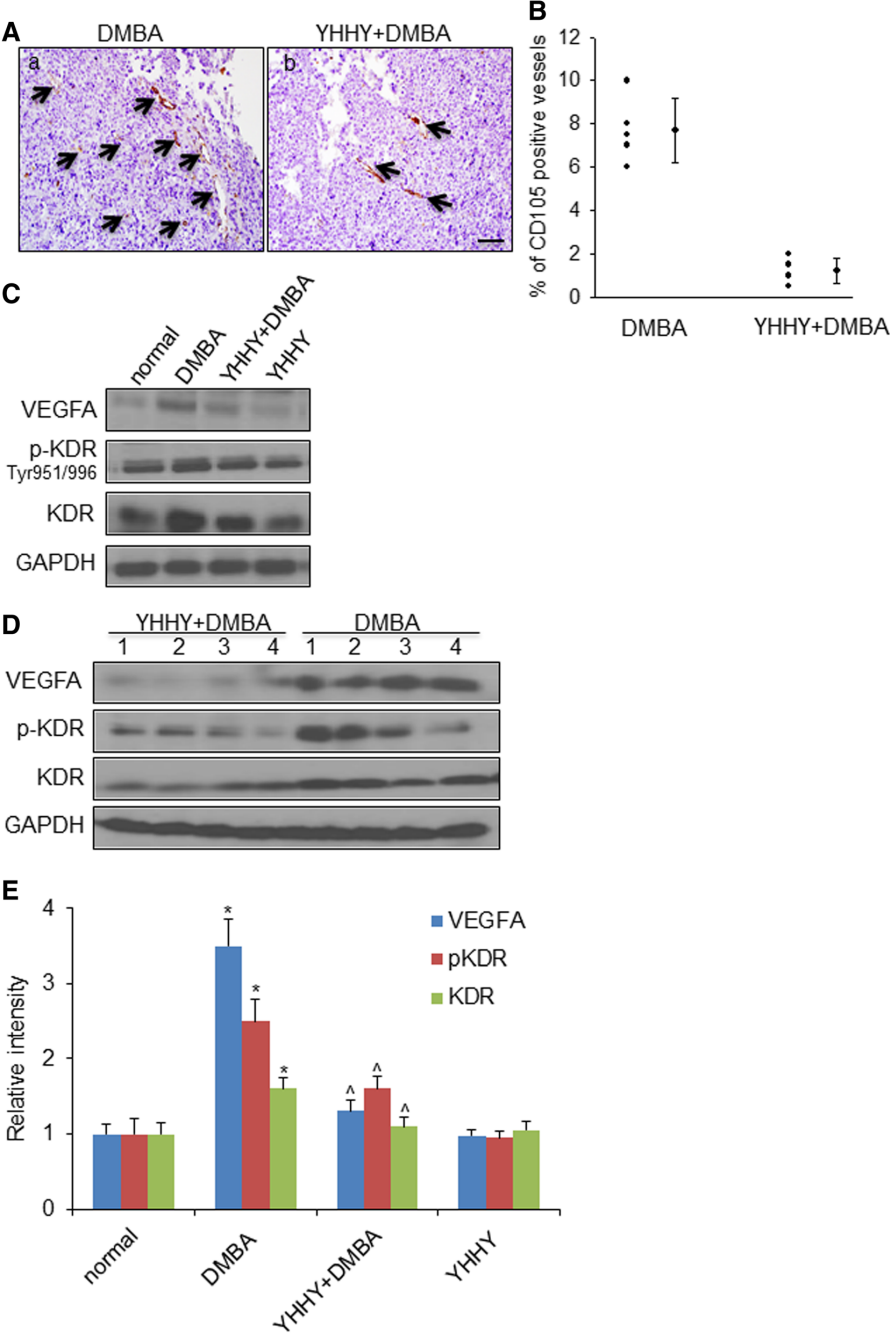
YHHY Decoction inhibited tumor neo-angiogenesis. **a**. Representative images of CD-105/Endoglin immunohistochemistry staining showing the newly formed blood vessels in tumor sections in a: DMBA-induced rat and b: YHHY treated DMBA rat. Black arrows point to the CD-105 positive blood vessels. Scale bar: 50 μm. **b**. Quantitative analysis of the vascular density in the tumor sections. *P* < 0.01, student t test. *n* = 5 animals per group. **c**. Representative Western blot images of VEGFA, p-KDR(Tyr 951/996) and total KDR in one tumor sample from each group. **d**. Expressions of VEGFA, p-KDR(Tyr 951/996) and total KDR in 4 individual tumors in the YHHY Decoction treated vs. DMBA control group. **e**. Quantification of the western blot analysis for VEGFA, p-KDR(Tyr 951/996) and total KDR in normal mammary glands (normal and YHHY groups) and mammary tumors (DMBA and YHHY+DMBA groups). * *p* < 0.05 vs. normal; ^ *p* < 0.05 vs. DMBA

The onset of angiogenesis is believed to be an early event in tumorigenesis. Like human breast pathology, the progression of DMBA-initiated tumor in the rat is associated with an angiogenic phenotype [27] (Fig. 4a). CD-105/Endoglin antibody was used here to label only newly formed blood vessels [49]. A remarkable reduction (80%) of CD-105/Endoglin positive microvessel density was observed in the YHHY Decoction-treated DBMA tumors in comparing with the control DMBA tumors (Fig. 4a, b).

Vascular endothelial growth factor (VEGF) and its receptor VEGFR2 or KDR play major roles in promoting the formation of new blood vessels [50, 51]. VEGFA and the receptor KDR were both examined to be expressed much lower in the YHHY treated tumors than those in the DMBA controls (Fig. 4c-e). While the less expressions of VEGF and its receptor may due to the less blood vessels in the YHHY treated DMBA tumors, we found that YHHY treatment blocked the activation of KDR at its major autophosphorylation site, i.e., Tyr951/996 [52] (Fig. 4c-e). These results suggested that the continuous use of YHHY Decoction in rats blocked the initiation and development of neoangiogenesis.

### YHHY decoction inhibited oxidative stress in the mammary tumors

The IPA analysis also revealed that regulation of free radical scavenging is one of the possible mechanisms of action of the YHHY Decoction. Numerous studies have shown that free radicals reactive oxygen species are generated by exposure to DMBA [53, 54]. A balance between free radicals and antioxidants is necessary for proper physiological function. If free radicals overwhelm the body’s ability to regulate them, a condition known as oxidative stress ensues. Free radicals thus adversely alter lipids, proteins, and DNA and trigger a number of human diseases including cancer [55]. Thus, the antioxidant capacity of YHHY Decoction was measured by two sets of analyses: (1) anti-oxidant enzyme activities of SOD, GPx and CAT; and (2) biochemicals of MDA and total NOx. As shown in Table. 3, DMBA induction significantly stimulated the enzyme activity of SOD and GPx in the mammary tumors by 54 and 61%, respectively (*P* < 0.01). Treatment with YHHY Decoction completely blocked the enzymatic activation, reduced the mean value of the SOD enzyme activity similar to those of the control group (*P* = 0.41) and an even lower of the GPx activity (*P* = 0.02). DMBA induction decreased the CAT activity by 17% in comparing to the control group (P < 0.01), and the application of YHHY Decoction had no further effect (*P* = 0.44). In addition, DMBA significantly increased MDA and NOx levels by 36 and 54%, respectively (P < 0.01), but these increases were completely blocked by YHHY Decoction for MDA (*P* = 0.47) and NOx (*P* = 0.38). These results indicated a strong capacity of YHHY Decoction on anti-oxidative stress.

**Table. 3:** Effects of YHHY Decoction treatment on antioxidant parameters in rats breast tissues

Group	SOD (U/mg protein)	GPx (U/mg protein)	CAT (U/mg protein)	MDA (nmol/mg protein)	NOx (μmol/mg protein)
(A) Raw data					
A-Control (*n* = 10)	2.68 ± 0.19	0.23 ± 0.02	624.5 ± 36.9	3.07 ± 0.37	5.84 ± 0.45
B-DMBA (*n* = 14)	4.12 ± 0.46	0.37 ± 0.06	520.1 ± 29.4	4.18 ± 0.42	8.99 ± 0.64
C-YHHY+DMBA (*n* = 18)	2.64 ± 0.33	0.19 ± 0.04	531.7 ± 31.7	3.02 ± 0.31	5.79 ± 0.47
D-YHHY (*n* = 10)	2.78 ± 0.12	0.24 ± 0.02	629.5 ± 29.2	3.19 ± 0.21	5.11 ± 0.39
(B) Statistical comparison					
Group A vs B	< 0.01	< 0.01	< 0.01	< 0.01	< 0.01
Group B vs C	< 0.01	< 0.01	0.44	< 0.01	< 0.01
Group A vs C	0.41	0.32	< 0.001	0.47	0.38
Group A vs D	> 0.05	> 0.05	> 0.05	> 0.05	> 0.05

*SOD* superoxide dismutase, *GPx* glutathione peroxidase, *CAT* catalase, *MDA* malondialdehyde, *NOx* nitrate

(A) Raw data given as mean ± S.E.M. (B) Statistical analysis (Tukey’s two-way comparisons) of the data shown in part A

## Discussion

The present study provides evidence for the first time that the ancient TCM herb cocktail YHHY Decoction exerts a notable chemopreventive activity in the experimentally-induced mammary tumorigenesis model, and more importantly, our bioinformatics and experimental approach systematically revealed the multi-components and multi-targeting mechanisms of the YHHY Decoction.

Our results indicate that daily oral intake of YHHY Decoction could significantly prevent or delay the development of mammary tumor in the rats. First, significantly lower percentage of YHHY-fed animals developed tumor within 16 wks after DMBA administration than the controls (61 vs. 94%, *P* = 0.041). Second, for the YHHY-fed rats that showed tumors, a much longer latent time was observed for the tumor development in compared to the controls (mean = 12.9 vs. 10.7 weeks, *P* = 0.015), and third, YHHY-treated tumors progressed much slowly than the control tumors within same time period, as manifested by a decreased tumor weight in the YHHY group (5.1 ± 1.7 vs. 19.5 ± 4.5, *P* < 0.05). These observations suggest that 1) the YHHY Decoction could substantially suppress the carcinogenic effect of DMBA; 2) the YHHY Decoction may selectively reverse the DMBA induced hyperplasia, thereby prohibit the development of breast cancer [13]. .

We observed suppressed myc activation by the YHHY decoction which may contribute to the prevention of tumorigenesis. Furthermore, the YHHY Decoction contains multiple bioactive components that work in a concert to modulate multiple dys-regulated pathways in precancerous cells and microenvironment (Table. 4). myc is one of the most potent oncogenes for cell transformation, but myc activation alone generally cannot induce tumorigenesis [47]. Tumorigenesis is a multifactorial process, in which carcinogenic factors disrupt the homeostatic molecular signaling networks, providing cells with essential physiological alterations to lead tumor formation. Sustained myc activation in a permissive epigenetic and/or genetic context orchestrates alterations of self-sustained growth, limitless replicative potential, angiogenesis, evasion of apoptosis, inflammation and oxidative stress [56–61]. These alterations are highly interconnected [56, 57]. For example, the carcinogen DMBA is demonstrated to induce high levels of oxidative stress in mammary gland [53, 54], subsequently induces overexpression of numbers of transcription factors (including c-myc) to promote the expression of genes related to cell proliferation, apoptosis and invasion [61]. To this end, the observed chemopreventive action of YHHY Decoction can be attributed to its capacity in regulating free radical scavenging and suppressing myc activation to potentiate tissue homeostasis.

**Table. 4:** Composition and targeted pathways of the YHHY Decoction in preventing DMBA-induced mammary tumorigenesis

Pinyin name	Botanical name	Examined chemical constituents	Targeted tumorigenic pathways
Lu Jiao Jiao	*Cornu Cervi Pantotrichum*	Adenosine Uracil	protect tissues against excessive inflammation and promote tissue repair
Tu Beimu	*Rhizoma Bolbostemmae*	tubeimoside A	inhibit proliferation; induce apoptosis
Bai Jie Zi	*Semen Brassicae*	allyl isothiocyanate	induce apoptosis; inhibit proliferation; anti-angiogenesis
Rou Gui	*Cinnamomum cassia*	cinnamic acid cinnamic aldehyde	antioxidant
Pao Jiang	*Baked Ginger*	6-shogaol	induce apoptosis; anti-angiogenesis
Ma Huang	*Ephdra Vulgaris*		
Hu Tao Rou	*Juglans regia L*		
Sheng Gan Cao	*Glycyrrhiza Uralensis*	Polysaccharides	induce apoptosis; antioxidant

Each component of the YHHY Decoction has been studied to regulate several different tumorigenic alterations, and several different components could regulate the same alteration pathways, which may implicate synergy [62, 63]. For example, 6-shogaol [64] has been shown to inhibit cancer cell growth and invasion, induce apoptosis and cancer cell differentiation, as well as suppress angiogenic factors [21, 65–67]. Cinnamic acid and cinnamic aldehyde have been identified with antioxidant, anti-inflammatory and cytotoxic properties [68]. Allyl isothiocyanate from *Semen brassicae* and tubeimoside A from *Rhizoma bolbostemmae* have been reported to inhibit tumor proliferation which was associated with cell cycle arrest and/or induction of apoptosis [23, 69, 70]. Allyl isothiocyanate also acts as an angiogenesis inhibitor in down-regulating VEGF and pro-inflammatory cytokines [71]. The polysaccharides from *Glycyrrhiza uralensis* exhibited antioxidant and immune-potentiation activities [40, 72]. Adenosine extracted from *Cornu Cervi Pantotrichum* can protect tissues against excessive inflammation and promote tissue repair [73]. Induction of apoptosis can be achieved by 6-shogaol, allyl isothiocyanate and tubeimoside A, same for antioxidant by cinnamic acid, cinnamic aldehyde and polysaccharides, and suppression of angiogenic factors by 6-shogaol and allyl isothiocyanate.

The IPA analysis helped us identify some key synergistic interactions occur on multiple tumorigenic signaling as many of the molecules interact with multiple components in the IPA database. For example, ERK1/2 is the interacting molecule with allyl isothiocyanate and adenosine, and Bcl2 is the interacting molecule with cinnamic aldehyde and allyl isothiocyanate. Although not all potentially bioactive constituents from the YHHY formula were identified, our study gained insight into the composition and mechanism by which YHHY Decoction abrogated mammary tumorigenesis in rats, and also demonstrated the holistic mode of action of the YHHY herb formula in targeting multiple systems.

Importantly, we didn’t observe any altered food intake, water intake, behavioral patterns, and growth rate of experimental animals upon YHHY treatment. This finding may indicate that the observed chemopreventive effect of YHHY Decoction is devoid of any toxic manifestation. Indeed, in comparing with synthesized chemical agents for anti-tumor growth, induction of apoptosis, anti-oxidant and anti-angiogenesis, the natural substances in the YHHY Decoction would be much less toxic [18]. Altogether, our study suggests that YHHY Decoction is a promising chemopreventive agent for breast cancer and merits further development.

## Conclusions

In conclusion, our study demonstrated that the ancient TCM herb cocktail YHHY Decoction exerted a striking chemopreventive activity in the experimentally-induced mammary tumorigenesis model. Our bioinformatics and experimental approach systematically revealed the multi-components and multi-targeting mechanisms of the YHHY Decoction. These data supports the use of YHHY Decoction as a beneficial chemopreventive drug in breast cancer.

## Abbreviations

ADH: Atypical ductal hyperplasia
ALH: Atypical lobular hyperplasia
*BG*: *Baked ginger*
CAT: Catalase
*CC*: *Cinnamomum cassia*
*CCP*: *Cornu Cervi Pantotrichum*
DCIS: Ductal carcinoma in situ
DMBA: 7,12-dimethylbenz(a) anthracene
*EV*: *Ephdra vulgaris*
GPx: Glutathione peroxidase
*GU*: *Glycyrrhiza uralensis*
H&E: Hematoxylin and eosin
HPLC: High performance liquid chromatography
*JRL*: *Juglans regia L*
LCIS: Lobular carcinoma in situ
MDA: Malondialdehyde
NOx: Nitrate
PAH: Polycyclic aromatic hydrocarbon chemicals
PBS: Phosphate-buffered saline
*RB*: *Rhizoma bolbostemmae*
*SB*: *Semen brassicae*
SD: Sprague-Dawley
SOD: Superoxide dismutase
TCM: Traditional Chinese Medicine
VEGF: Vascular endothelial growth factor
YHHY: Yanghe Huayan Decoction
